# Vaccination

**Published:** 1901-08

**Authors:** Joseph Grindon

**Affiliations:** St. Louis, Mo.


### VACCINATION.*

By JOSEPH GRINDON, M.D.,

ST. LOUIS, MO.

One might imagine that in this the first year of the Twentieth Century there would be little excuse for writing a paper on vaccination. Jenner's discovery is more than a hundred years old and

* Read before the Medical Society of City Hospital Alumni, February 27, 1901.

its value was acknowledged from the first by all the best minds of the profession. True, testimony to its value is constantly accumulating and some new facts concerning it—symptomatic, pathologic and histologic, have from time to time come to light, but I shall have nothing to offer to-night which is not already accessible to you all.

What then is my excuse for choosing this subject? First, that the repetition of the best-known truths is not useless, and that it is well to be occasionally reminded of the reasons for the faith that is in us.

Second, that among that larger audience which I may hope to reach through you, there are still some who do not appreciate the value of this, among the greatest blessings vouchsafed to humanity. Proof of this assertion is easily furnished by the fact that during this and last winter smallpox has been rife throughout the United States, whereas a century ago it seemed probable that it would soon be stamped out from the face of the civilized world. Every pitted face is a reproach to civilization.

Third, because even among medical men the science and art of vaccination are but ill understood. Pus infections and other spurious inoculations still occasionally pass for vaccinations, leaving their recipients worse off than before, because in fancied and unreal security. Even true vaccinations are in large proportions imperfect and insufficient, and thus undeserved doubt is thrown upon the efficacy of the procedure. The records of all smallpox hospitals show a certain proportion of patients bearing one or more vaccine scars. This should not be, and would not be, did all vaccinators thoroughly understand their business.

The course of vaccinia in the human subject presents a close parallel to that of variola, which is not surprising when we remember that in fact it *is* variola, modified by its passage through the organism of the cow.

Usually at the close of the third day after vaccination a slight elevation of the abraded surface may be seen; this is better marked the next day. By the fifth day the epidermis begins to be pushed up by a flaccid accumulation beneath, and on the sixth day a distinct multilocular, bluish-white vesicle has been formed, with raised edges and a saucerlike central depression. The vesicle continues to spread at its periphery for two days more, when, that is on the eighth day, a specific imflammation, marked by a red circumferential area, the areola, makes its appearance. The vesicle and areola now present the appearance beautifully described by Jenner as "the pearl on the rose." It is the formation of this areola which signalizes the event of systemic intoxication. It is the index of immunity. From that moment, and not before, is the individual vaccinated. For two days more and until the tenth day both vesicle and areola enlarge; the latter may now be two inches or more in width, and is of a dusky hue. The arm is hot, swollen and tender; the inflammation has extended to the neighboring subcutaneous connective tissue. Contiguous lymphatic glands may be implicated; there is more or less systemic disturbance, fever, anorexia, restlessness, etc.

After the tenth or eleventh day, the pustule, for such it has now become, begins to dry in the center, the areola grows narrower and the local and constitutional symptoms begin to abate. By the fourteenth or fifteenth day a dark mahogany colored scab has formed, which falls off between the seventeenth and twenty-fifth day.

The course of events may vary somewhat from what has just been stated; the variation may be either in the way of *acceleration*, as when long-humanized virus is used, or in the way of *retardation*, as is likely to be the case with bovine virus.

This retardation may occur, (1) during incubation; this may occur with any dry lymph, but more particularly the bovine, owing to its insolubility. (2) During desiccation and desquamation, the scab, in fact, occasionally remains attached for many weeks.

The resulting cicatrix is at first vascular but soon becomes white, and should present a foveated and somewhat radiated appearance.

The test for vaccination, which has received the name of Bryce, consists in reinserting the virus on the fourth, fifth or sixth day after primary vaccination, when, if the first have been successful, the vesicles of the second insertion are hurried forward so that all come to maturity on the same day. The vesicles and areola of the second insertion are much smaller than those of the first. If there be no acceleration of the second lot, the first is supposed to have failed, and then the second is to be regarded as the initial vaccination and to be tested by a third, and so on.

It is important that one should be informed as to the existence and clinical characters of the several forms of spurious vaccinia recognized and described. It is unnecessary to dwell upon the possible sinister effects of an error of diagnosis here, through which an individual may suppose himself protected when in reality his susceptibility to smallpox is as great as ever. The various forms are:

1. Red tubercles, the size of peas, which afterward suppurate. This form was very common in St. Louis some years ago, and has been observed by me within the last year. The little tumors may persist for weeks.

2. A non-umbilicated, but acuminated or conoidal vesicle, commencing with much irritation and itching, containing straw-colored or opaque, instead of clear, lymph. The areola is completed on the fifth or sixth day, beginning to decline on the eighth day and the scab falls off by the tenth.

3. Instead of the usual vesicle, a bleb sometimes forms, followed by troublesome ulceration.

4. A crop of herpetic vesicles, preceded by shivering and accompanied by intolerable itching and enlargement of axillary glands. The bursting of the vesicles is followed by an eczematous inflammation.

5. Occasionally vesicles which have apparently run a normal course up to the eighth or tenth day, suddenly rupture and are followed by ulceration which extends both in depth and peripherally; three such cases I observed recently. These cause much local and constitutional disturbance. Of course, any of these events call for revaccination.

Among the possible complications are inflammation and suppuration of neighboring and even of distant lymphatic ganglia. My personal opinion is that this is always due to a filthy vaccination.

Various cutaneous manifestations may accompany the constitutional disturbance incident to vaccinia. The most common of these is a roseola identical in appearance with that at times accompanying dentition. Papular eruptions, urticaria, erythema multiforme, purpura and zoster have also been recorded. Vesicular and bullous eruptions may occur, at times exactly copying the vesicular and bullous types of dermatitis herpetiformis. A number of such cases were reported to the American Dermatological Association at its meeting in 1900 by Drs. Bowen of Boston, Pusey of Chicago, Dyer of New Orleans, myself and others. In my two cases the eruption appeared—as seemed the rule—about four weeks after vaccination. Large tense blebs arranged in groups and circles were scattered over the general body surface in a symmetrical fashion. Some of the Boston cases had existed for a year or more when reported. Still graver are the cases reported by Hutchinson and Stokes under the name of vaccinia gangrenosa. There appeared purpuric spots which later became gangrenous. Fatalities have been known.

Serious as these well-established cases are, they are so few as to sink into insignificance when compared to the immense number benefited by vaccination.

Vaccination may determine the appearance of eczema in those predisposed to it, or aggravate it when it already exists. For this reason it may be well to defer the operation in some cases, provided, of course, that there has been no exposure to smallpox.

When one of the continued fevers makes its appearance before the eighth day of vaccination, that is, before the appearance of the areola, it will sometimes happen that the progress of the vaccinia will be arrested until the subsidence of the more important disease, when it will take up the thread of its tale where it was dropped. At other times the two diseases will go on together. Of the relations of this sort existing between vaccinia and variola I shall speak later.

Revaccination is second in importance only to primary vaccination. Its course departs more or less from that of vaccination. The vesicles may reach their acme by the fifth, fourth or third day, sometimes the process does not go beyond papulation.

The question is frequently asked, How often should one be revaccinated? We may first observe that it seems to be a fact that any vaccinal impress may wear out in time. In *what* time, depends upon the nature of the first vaccination, the time of life at which it was done, and individual peculiarity.

I obtained a record of the date of last vaccination in 555 cases of smallpox observed by myself, with the following result:

| | CASES. | DEATHS. | PER CENT. |
|---|---|---|---|
| Never vaccinated | 314 | 146 | 46.5 |
| Vaccinated over 15 years | 123 | 29 | 23.5 |
| Vaccinated between 10 and 15 years | 23 | 6 | 26.0 |
| Vaccinated between 5 and 10 years | 27 | 5 | 18.5 |
| Vaccinated within 5 years | 68 | 11 | 16.0 |
| Total | 555 | 197 | 35.7 |

This table shows not only the protecting influence of vaccination but its power in mitigating the severity of the disease.

As to the time of life, puberty and the changes incident thereto lessen the potency of the vaccinal impress received at an earlier date. For that reason vaccination should be again resorted to soon after.

The following table, taken from Corry, shows the gradual effacement of the vaccinal impress, at the present day, and also how, without vaccination, smallpox is eminently a disease of early life as are the other exanthemas, and for the same reason:

PERCENTAGES OF DEATH FROM SMALLPOX AT VARIOUS AGES.

| | Under 5 | 5–20 | 20–40 | 40–60 | 60–80 | Over 80 |
|---|---|---|---|---|---|---|
| Pre vaccination times | 83.15 | 15.79 | 1.16 | 0 | 0 | 0 |
| Present | 3.07 | 16.34 | 58.41 | 18.61 | 3.24 | .32 |

Trousseau's rule, that one should be vaccinated every five years, while probably an excess of precaution, is a good one. At all events, one should revaccinate whenever there is danger of infection.

*Superiority of Bovine Virus.*—Now, as to the kind of virus one should use. There are certain facts which should guide us in our choice. Virus many removes distant from its fountain head, that is, which has been often transmitted since first taken from the cow, has lost much of its prophylactic power.

Four years after Jenner had given his discovery to the world, a committee of the House of Commons, appointed for the purpose, could only find two cases of post-vaccinal smallpox. In Copenhagen, at the time a city of 100,000 souls, where vaccination was rigidly enforced, not a single fatal case of smallpox occurred during the thirteen years ending with 1823. At Annspach, Bavaria, a place of 300,000 inhabitants, not a fatal case occurred during the nine years ending with 1818. Of over 500,000 vaccinated in France between 1804 and 1813, only seven are known to have taken smallpox.

Soon, however, it began to be apparent that the beneficent influence of the discovery was on the wane. In France, between 1819 and 1835, there were 5,467 cases of post-vaccinal smallpox, of which 51 were fatal. In Copenhagen, there were several epidemics between 1825 and 1835, in which there were 3,093 post-vaccinal cases and 66 deaths.

There is, however, a place for the use of humanized virus, namely, in cases showing relative insusceptibility to vaccinia. Facts seem to show that failures are rarer when humanized virus is used than when bovine. Thus Corry reports in his own practice failures in 311 first vaccinations with bovine and one in 1,140 with humanized virus, using in both instances the fresh unstored lymph, that is, arm to arm or calf to arm.

The formula of insusceptibility is expressed as follows: Let X represent the number of primary vaccinations yielding one failure. Now, if the probability of success was the same for a second or a third vaccination as for the first, the ratio of failures for third attempts would be 1 to $X^3$. The fifth and sixth volumes of "Reports of the New York State Board of Health," however, show that failures are more frequent than 1 to $X^3$. That is, that one in whom a vaccination has failed is less likely to have a success than one never vaccinated, the ratio of failure being $\frac{2X^3}{9}$. Say that the ratio of failure for first attempt is 1 to 300, then $\frac{2(27,000,000)}{9}=6,000,000$.

*Selection of the Subject.*—Cutaneous and intestinal affections contraindicate vaccination; chronic diseases—such as syphilis and struma—offer no impediment, neither does pregnancy. Of course, in the face of exposure to smallpox all ordinary rules should be set aside and the individual rendered as nearly immune as possible with the least loss of time.

The best age at which to vaccinate is three months, that is before the commencement of the troubles incident to dentition. Of course, when smallpox prevails, the child should be vaccinated at birth. I have seen a child sicken with smallpox on the tenth day of its life; the mother was well; it must have inhaled the germ almost with the first lungful of air. This may serve to impress the moral of early vaccination.

The question may be asked: Is there any use in vaccinating persons who have been exposed to smallpox for a day or more? I desire to give an answer in no uncertain voice. Yes, of course, and without the loss of a single precious moment. First, because you cannot tell when the germ enters; one may resist the infection to-day and receive it to-morrow. Second, because even though one be already infected, it may still be in time to save health, or, if not that, perhaps life. The average duration of incubation of smallpox may be called thirteen days. The areola of vaccination marking the reception of the systemic impress occurs on the eighth or ninth day. This gives us three days or so in which to work out the salvation of the threatened individual. If not in time to prevent the disease altogether, it may be in time to mitigate it.

Marson states the matter as follows: Suppose an unvaccinated person to inhale the germ of variola on a Monday, if he be vaccinated as late as the following Wednesday the vaccination will be in time to prevent smallpox being developed; if it be put off until Thursday the smallpox will appear, but will be modified; if the vaccination be delayed until Friday it will be of no use, it will not have had time to reach the stage of areola, the index of safety, before the illness of smallpox begins.

